# Fall risk, fall awareness, and social support among 825 community—dwelling older adults with functional limitations: a cross—sectional study

**DOI:** 10.3389/fpubh.2026.1763697

**Published:** 2026-02-25

**Authors:** Li Xiao, Rong Hu, Xiaocui Zou, Hong Wu

**Affiliations:** Sichuan Provincial People’s Hospital, University of Electronic Science and Technology of China, Chengdu, China

**Keywords:** community-dwelling older adults with functional limitations, cross-sectional study, fall risk, self- awareness, social support

## Abstract

**Purpose:**

This study aimed to examine the level of fall risk and its associations with fall awareness and social support among community-dwelling older adults with functional limitations.

**Methods:**

A cross-sectional study was conducted from January to October 2025. Using cluster sampling, 825 older adults with functional limitations were recruited from four communities in Chengdu, China. Fall risk was assessed using the Modified Falls Risk for Older People in the Community Assessment (MFROP-com), fall awareness was measured with the Self-awareness of Falls in Elderly Scale (SAFE), and social support was evaluated via the Social Support Rating Scale (SSRS). Data were analyzed using Pearson correlation and multiple linear regression.

**Results:**

Participants exhibited a high fall risk (mean score 26.51 ± 10.95). The regression model explained a significant proportion of variance in fall risk (Adjusted *R*^2^ = 0.536, *p* < 0.001). Higher fall awareness (B = −0.463, *β* = −0.565, *p* < 0.001) and greater social support (B = −0.422, *β* = −0.190, *p* < 0.001) were independent predictors of lower fall risk. Advanced age, unmarried, living alone, and having sleep disorders were associated with increased risk (*p* < 0.05).

**Conclusion:**

Fall awareness and social support were identified as key modifiable factors associated with reduced fall risk among community-dwelling older adults with functional limitations. Fall prevention programs should focus on enhancing risk perception and strengthening social support networks for this population.

## 1 Introduction

Population ageing is a defining trend of our time. It is projected that by 2050, 80% of the world’s older population will reside in low- and middle-income countries, where the most substantial demographic shifts are occurring (1). As a prominent example, China, a typical developing country, is experiencing rapid growth of its older population (2). Within this group, older adults with functional limitations represent a critical sub-population, numbering 40.63 million and constituting 18.3% of all seniors (3). Compared to the general ageing population, this group exhibits poorer health status and faces greater health risks, making them a priority for safety care (4).

In China, community-dwelling older adults represent a core segment of the ageing population (5). Their preference for “ageing in place” (6) is strongly shaped by traditional cultural values, notably the concept of “returning leaves to their roots” and the Confucian ethic of “filial piety” (7). Moreover, living within a community helps older adults maintain family bonds and receive a certain amount of social support through the effective mobilization of family and community resources. Nevertheless, this preferred living arrangement also entails significant risks, with falls being a primary concern for them. The elevated fall risk among community-dwelling older adults stems from multiple factors, including hazardous community environments, insufficient access to professional care, and a general lack of fall awareness.

A fall is defined as “an unintentional loss of balance, in which the individual comes to rest on the ground, floor, or lower level” (ICD-10 codes W00–W19). As a common health and safety incident, falls rank as the sixth leading cause of death among individuals aged 65 and above (8). Statistically, Falls in the older population typically occur in domestic or community settings (9). For community-dwelling older adults, the annual incidence of falls ranges from 14.3% to 19.3% (10), with up to 79.50% resulting in injury (11). This risk is markedly elevated among older adults with functional limitations, who experience falls more frequently, suffer more severe consequences, and are prone to a debilitating “fall–reduced activity–functional decline” cycle (12). Studies indicate that the objectively detected fall risk in this population ranges from 41.3% to 67.97% (13, 14). However, their self-assessed fall risk rate is considerably lower (29.2%–34.8%) (15). This significant discrepancy between objective risk and subjective perception exists a pervasive lack of fall awareness in this high-risk group.

Fall awareness refers to an older adult’s subjective assessment of their own risk of falling (16). This concept encompasses personal evaluations of both the likelihood and potential severity of a fall incident (17). Within the cognitive-behavioral theory, fall awareness acts as a mediating variable, significantly influencing an individual’s decision to adopt fall preventive measures (18). It also elucidates why even those in high - risk categories frequently demonstrate insufficient participation in fall prevention initiatives from the perspective of individual self - awareness (19, 20). Consequently, weak fall awareness leads to inadequate protective behaviors and poor adherence to prevention strategies, thereby substantially increasing the risk of falling (21).

Social support encompasses emotional concern from family members, physical assistance from caregivers, and environmental enhancements combined with community-based health education (22). Scholar indicates that adequate social support can reduce fall risk among older adults (23). For instance, family caregivers can bolster older adults’ vigilance and self-efficacy regarding falls through reminder, companionship, and exercise assistance (24). Similarly, healthcare workers promote realistic self-assessment by conducting risk evaluations and providing health education, which in turn increases fall awareness and motivates behavioral change (25). From a social support perspective, Community-dwelling older adults with functional limitations can benefit from familial emotional concern and optimally utilize family and community resources. Therefore, social support may act as a direct promoter of their fall prevention behaviors and as a psychological empowerment pathway that indirectly strengthens their fall awareness (26, 27).

Current research on fall risk primarily focuses on hospitalized or institutionalized older adults (16, 28), while community-dwelling older adults with functional limitations have received less attention. Moreover, existing work has predominantly focused on physiological risk factors, overlooking the older adults as holistic entities and neglecting a comprehensive assessment of psychological and social factors associated with falls (29, 30). Therefore, this study aims to assess fall risk, fall awareness, and social support among this population and to examine their interrelationships. The findings are expected to provide an empirical basis for developing community-based fall prevention interventions that enhance awareness and social support, thereby reducing falls incidence and promoting healthy aging.

## 2 Materials and methods

### 2.1 Study population and design

This study adopted a descriptive cross-sectional design, which was conducted from January 13 to October 31, 2025. Participants were selected by a cluster sampling method and came from four communities in Chengdu, Sichuan Province, China. The Chengdu city comprises 14 suburban counties, known as Longquanyi, Wenjiang, Xindu, Pidu, Shuangliu, Qingbaijiang, Jintang, Pujiang, Xinjin, Dayi, Dujiangyan, Pengzhou, Chongzhou, and Qionglai. The city is divided geographically into four regions: East, South, West and North, with one suburban district randomly selected from each region.

Older adults with functional limitations, measured by the Barthel Activities of Daily Living Index (Barthel ADL Index) (31). The Barthel ADL Index was obtained by asking 10 questions to assess an individual’s functional capacity. The higher the score, the better the performance of ADL. With scores ranging between 0 and 100 points, different degrees of functional limitation were established: severe functional limitation (0–40 points), moderate functional limitation (41–60 points), and slight functional limitation (61–99 points) (32).

Inclusion criteria comprised: (1) Chinese community-dwelling older adults, aged ≥60 years; (2) Older adults with functional limitations: Barthel ADL Index < scored 100 points, which was assessed by trained researchers. (3) Ability to communicate with the researchers and cooperate with the physical assessment tester; and (4) Willingness to participate in the research. Exclusion criteria comprised: (1) Being in the acute phase of any disease (e.g., acute myocardial infarction, stroke, infection, or major surgery within the past 3 months) that significantly impaired the ability to participate in assessments or communicate stably; or (2) Being in the terminal phase of a disease, as determined by a physician’s diagnosis with an expected survival of less than 6 months.

### 2.2 Sample size

The sample size was determined *a priori* using G*Power 3.1. An *F*-test for multiple linear regression (fixed model, *R*^2^ deviation from zero) was selected. In the absence of prior studies for estimating the expected *R*^2^, we defined a minimum effect size of interest (MESOI) based on Cohen’s conventions (33). A population *R*^2^ of 0.13 (medium effect size, *f*^2^ = 0.15) was chosen as the MESOI. With *α* = 0.05, power = 0.80, and *k* = 18 predictors, the analysis indicated a minimum sample size of *n* = 150. To accommodate potential missing or incomplete data in questionnaires, we aimed to recruit at least 180 participants.

As data were collected across multiple centers, we conducted expanded recruitment to ensure sufficient statistical representation within the stratified subgroups of each geographic community. Furthermore, during the projected data collection period, the response rate was significantly higher than anticipated. Ultimately, 825 valid responses were collected.

The enlarged sample enhanced the statistical power (>0.99) and the stability of estimates, facilitating more reliable detection of effects for key variables. We acknowledge that with this large sample size, even effects of small magnitude may reach statistical significance. Therefore, in our analysis, we emphasized the interpretation of effect sizes to avoid relying solely on *p*-values.

### 2.3 Measurements

#### 2.3.1 Participants’ general characteristics

General characteristics were selected based on a comprehensive literature review and synthesized clinical expertise. A self-designed questionnaire was used to collect participants’ information. This included: (1) socio-demographic factors: sex, age, marital status, education level, monthly income, number of children, living arrangement, and etc.; (2) functional health: degrees of functional limitation and walking aids usage; (3) psychological factors: fear of falling, knowledge of fall prevention, and frequency of negative emotions;(4) health status indicators: sleep quality and nutritional status.

#### 2.3.2 Modified falls risk for older people in the community assessment

This study employed the Falls Risk for Older People in the Community (FROP-com) scale to assess modifiable fall risk factors in older adults (34). Originally developed by the Australian Institute of Ageing in 2008, the scale was later translated and validated for Chinese populations by Wang Lwei in 2011 (35). The Chinese Modified version (MFROP-com) consists of 13 items comprising 19 specific assessments. Six items were scored on a binary scale (0–1 points), while the remaining seven items use a four-point ordinal scale (0–3 points). Total scores range from 0 to 45, with higher scores indicating increased risk of falls. Fall risk levels were categorized as: 0 (no risk), 1–12 (mild risk), and >12 (high risk). The scale has shown good internal consistency, with a reported Cronbach’s *α* of 0.804 in the original validation study. In the current study, Cronbach’s *α* was 0.869.

#### 2.3.3 Self-awareness of falls in the elderly scale

The Self-awareness of Falls in Elderly scale (SAFE), developed by Meei-Ling Shyu in Taiwan, China, was used to assess older adults’ self-awareness of fall risk (16). This 21-item scale covers four dimensions: (1) awareness of activity safety and environment (8 items); (2) awareness of physical functions (6 items); (3) awareness of medication (3 items); and (4) awareness of cognitive behavior (4 items). All items were rated on a 5-point Likert scale. For 15 items, scores range from 1 (“strongly disagree”) to 5 (strongly agree”). The remaining 6 items (items 1, 4, 6, 7, 8, and 15) are reverse-scored. The total score ranged from 21 to 105, with higher scores indicating greater awareness of fall risk. The scale has demonstrated strong reliability and validity in Chinese institutionalized and hospitalized older adults, with a previously reported Cronbach’s *α* of 0.923. In this study, its Cronbach’s *α* was 0.898.

#### 2.3.4 The social support rating scale

The Social Support Rating Scale (SSRS) was used to measure participants’ perceived social support. This instrument, widely used in China, was originally compiled by scholar Xiao Shuiyuan in 1986 (36). The scale consists of 10 items across three dimensions: (1) subjective social support (4 items), (2) objective social support (3 items), and (3) utilization of social support (3 items). Items are rated on a 4-point Likert scale. Total scores range from 12 to 66, with higher scores indicating a greater perceived adequacy of social support. Scores are categorized as low (≤22), moderate (23–44), and high (45–66). The SSRS has demonstrated strong internal consistency. Cronbach’s *α* was 0.913 in the original validation study and 0.867 in the present study.

#### 2.3.5 The visual analogue scale for fear of falling

The Visual Analogue Scale for Fear of Falling (VAS-FOF) was used to measure older adults’ fear of falling (37). The VAS-FOF ranges from 1 to 10, where 1 indicates no fear and 10 indicates extreme fear. Participants selected the number that best reflected the intensity of their fear of falling. In this study, a score of 1 was classified as no fear of falling, while scores from 2 to 10 were considered to indicate the presence of fear of falling.

#### 2.3.6 Chinese version of the positive and negative affect scale

The Positive and Negative Affect Scale (PANAS) was developed by Watson et al. in (38), which comprises two sub-scales measuring the frequency of positive and negative emotions. For the present study, we used the Chinese version of PANAS revised by Huang to assess participants’ negative emotions. Responses were recorded on a 5-point scale (1 = very slightly or not at all, 2 = a little, 3 = moderately, 4 = quite a bit, 5 = extremely) (39). Participants indicated how frequently they had experienced each emotion over the past few weeks.

### 2.4 Data collection

This study used a questionnaire survey. Participants were mainly recruited and surveyed at community centers—including neighborhood committee offices and senior activity centers—within the four selected communities. Although most questionnaires were self-administered, interviewers assisted participants with limited literacy to ensure accurate completion. Questionnaires were collected immediately upon completion. To ensure data integrity, investigators carefully reviewed each questionnaire for completeness. Any missing were identified, and respondents were asked to clarify them.

Out of 850 questionnaires distributed, 825 valid responses were obtained, yielding a response rate of 97.1%. To ensure accuracy, two assistants independently entered all data; discrepancies were resolved through comparison. Data cleaning included checks for logical consistency, out-of-range values.

### 2.5 Statistical analysis

All statistical analyses were performed using SPSS (version 23.0; IBM Corp., Armonk, NY, USA). Categorical variables are presented as numbers and percentages. Continuous variables were tested for normality and are expressed as mean ± standard deviation (M ± SD). For dichotomous variables (e.g., gender), independent samples *t*-tests were used. For categorical variables (e.g., age groups, living arrangement), comparisons were performed using one-way analysis of variance (ANOVA). If ANOVA results were significant, further pairwise post-hoc comparisons were conducted. Associations between fall risk, fall awareness, and social support were examined using the Pearson correlation analysis. Multiple linear regression was performed to identify factors associated with fall risk, incorporating significant socio-demographic and fall-related variables as independent predictors and the score of MFROM-com as the dependent variable. A two-sided *p*-value < 0.05 was considered statistically significant in this study.

## 4 Results

### 4.1 Characteristics of community-dwelling older adults with functional limitations

A total of 825 community-dwelling older adults with functional limitations were included (mean age = 75.42 ± 8.65 years; 51.27% male). The vast majority (90.42%) had slight functional limitation. Living arrangements varied, including living alone (27.03%), with a spouse (26.30%), or with relatives (28.12%). The overall fall risk was high (MFROP-com score: 26.51 ± 10.95). Participants demonstrated a moderate level of fall awareness (SAFE score: 57.89 ± 13.35) and perceived social support (SSRS score: 46.44 ± 4.94). The complete distribution of all socio-demographic and clinical characteristics is presented in Table 1.

**Table 1:** Participants’ characteristics and univariate analyses (*n* = 825).

Variables	Total *n* (%)	Fall risk (M ± SD)	*F/t*	*P*
Sex			*t* = −0.869	0.385
Male	423 (51.27)	26.83 ± 10.67		
Female	402 (48.73)	26.17 ± 11.23		
Age (years)			*F* = 10.596	<0.001**
60 ~ 69	236 (28.61)	24.79 ± 10.62		
70 ~ 79	323 (39.15)	25.75 ± 10.07		
≥80	266 (32.24)	28.96 ± 11.84		
Marital status			*F* = 16.123	<0.001**
Unmarried	131 (15.88)	32.17 ± 7.06		
Divorced	155 (18.79)	26.88 ± 11.75		
Widowed	176 (21.33)	25.63 ± 10.84		
Married	363 (44.00)	24.74 ± 11.14		
Education level			*F* = 2.961	0.031*
Primary school and below	384 (45.82)	27.72 ± 10.72		
Secondary school	332 (41.82)	25.52 ± 10.55		
High school	24 (2.91)	24.88 ± 11.50		
College and above	85 (9.45)	25.36 ± 12.81		
Monthly income (CNY)			*F* = 0.159	0.924
<1,000	160 (19.39)	26.88 ± 11.09		
1,000–2,999	238 (28.85)	26.60 ± 10.47		
3,000–4,999	214 (25.94)	26.11 ± 11.31		
≥5,000	213 (25.82)	26.53 ± 11.05		
Number of children			*F* = 1.920	0.147
0	71 (8.61)	28.38 ± 11.41		
1	87 (10.55)	27.71 ± 10.83		
≥2	667 (80.85)	26.15 ± 10.90		
Living arrangement			*F* = 32.273	<0.001**
Living alone	223 (27.03)	28.48 ± 11.38		
Living with a spouse	217 (26.30)	20.61 ± 8.03		
Living with relatives	232 (28.12)	29.24 ± 11.13		
Living with care worker	153 (18.55)	27.87 ± 10.58		
Degrees of functional limitation			*F* = 4.587	0.010*
Slight functional limitation	746 (90.42)	26.84 ± 10.87		
Moderate functional limitation	61 (7.39)	24.39 ± 11.21		
Severe functional limitation	18 (2.18)	20.11 ± 11.13		
Walking aid usage			*t* = −1.554	0.121
Yes	140 (16.97)	25.20 ± 10.89		
No	685 (83.03)	26.78 ± 10.95		
Fear of falling			*t* = 0.503	0.615
Yes	274 (33.21)	26.24 ± 10.95		
No	551 (66.79)	26.64 ± 10.95		
Knowledge of fall prevention			*F* = 8.491	<0.001**
Understand	257 (31.15)	24.93 ± 10.34		
Fair understanding	319 (38.79)	28.44 ± 10.96		
Do not understand	249 (30.06)	25.67 ± 11.21		
Frequency of negative emotions			*F* = 2.230	0.064
Very slightly or not at all	160 (19.39)	25.67 ± 11.21		
A little	183 (22.18)	26.45 ± 11.25		
Moderately	159 (19.27)	25.30 ± 10.67		
Quite a bit	175 (21.21)	25.59 ± 11.15		
Extremely	148 (17.94)	28.64 ± 11.24		
Sleep quality			*t* = −4.913	<0.001**
No sleep disorder	119 (14.42)	21.56 ± 12.08		
Sleep disorder	706 (85.58)	27.34 ± 10.53		
Nutritional status (BMI, kg/m^2^)			*F* = 1.660	0.191
Normal (18.5–23.9)	615 (74.55)	26.42 ± 10.94		
Underweight (<18.5)	109 (13.21)	25.49 ± 10.94		
Overweight/obese (≥24)	101 (12.24)	28.17 ± 10.90		

**Correlation is significant at the 0.01 level (2-tailed); *Correlation is significant at the 0.05 level (2-tailed).

BMI, body mass index.

### 4.2 Comparison of demographic variables across the fall risk among community-dwelling older adults with functional limitations

One-way ANOVA and t-tests were used to analyze continuous variables and compare differences in demographic characteristics by fall risk. Univariate analysis results were summarized in Table 1 and revealed the following: participants aged 80 years and above scored significantly higher than those aged 70–79 or 60–69 years (*F* = 10.596, *p* < 0.001); Unmarried participants scored significantly higher than those of other marital statuses (*F* = 16.123, *p* < 0.001). Those with a primary school education or below scored significantly higher than those with higher education levels (*F* = 2.961, *p* = 0.031). Participants living with a spouse scored significantly lower than those living with others (*F* = 32.273, *p* < 0.001). Participants with slight functional limitation scored significantly higher than those with moderate or severe functional limitation (*F* = 4.587, *p* = 0.010). Both knowledge of fall prevention (*F* = 8.491, *p* < 0.001) and sleep quality (*t* = −4.913, *p* < 0.001) were risk factors associated with increased scores.

### 4.3 The fall risk, self-awareness of falls, and social support among community-dwelling older adults with functional limitations

The mean scores for community-dwelling older adults with functional limitations were 26.51 ± 10.95 for fall risk, 57.89 ± 13.35 for fall awareness, and 46.44 ± 4.94 for social support. Table 2 presents the total scores and per item scores for each scale and its sub-dimensions. Total scores reflect the overall level of each construct, while per item scores allow comparison of response intensity across sub-scales with differing numbers of items.

**Table 2:** Scores of MFROP-com, SAFE, and SSRS among participants (*n* = 825).

Dimension	Total score(M ± SD)	Mean score(M ± SD)
MFROP-com	26.51 ± 10.95	1.40 ± 1.21
SAFE	57.89 ± 13.35	2.76 ± 1.64
Awareness of activity safety and environment	24.12 ± 6.46	3.02 ± 1.54
Awareness of physical functions	11.20 ± 4.99	1.87 ± 1.29
Awareness of medication	9.70 ± 4.36	3.23 ± 1.70
Awareness of cognitive behavior	12.88 ± 6.09	3.22 ± 1.74
SSRS	46.44 ± 4.94	3.32 ± 1.51
Objective social support	11.44 ± 3.60	3.81 ± 2.52
Subjective social support	24.08 ± 2.73	3.01 ± 1.07
Support utilization	10.92 ± 1.95	3.64 ± 0.81

MFROP-com, modified falls risk for older people in the community assessment; SAFE, self-awareness of falls in elderly scale; SSRS, the social support rating scale.

### 4.4 Correlation among fall risk, fall awareness, and social support

Pearson’s correlation analysis revealed a significant negative correlation between the total score on the MFROP-com and the SAFE (*r* = −0.680, *p* < 0.01). The MFROP-com total score was also negatively correlated with all SAFE sub-dimensions: Activity Safety and Environment (*r* = −0.551, *p* < 0.01), Physical Function (*r* = −0.395, *p* < 0.01), Medication Safety (*r* = −0.411, *p* < 0.01), and Cognitive Behavior (*r* = −0.287, *p* < 0.01). Furthermore, the MFROP-com total score showed a significant negative correlation with the SSRS total score (*r* = −0.348, *p* < 0.01). The negative correlation with subjective social support was stronger (*r* = −0.411, *p* < 0.01), while the negative correlation with objective social support was weaker (*r* = −0.160, *p* < 0.05). Conversely, the SAFE total score showed a significant positive correlation with the SSRS total score (*r* = 0.227, *p* < 0.01). Detailed results are provided in Table 3.

**Table 3:** Correlation analysis of MFROP-com, SAFE, and SSRS (*n* = 825, *r* values).

Variable	MFROP-com	SAFE	S1	S2	S3	S4	SSRS	SS1	SS2	SS3
MFROP-com	1									
SAFE	−0.680**	1								
S1	−0.551**	0.694**	1							
S2	−0.395**	0.591**	0.234**	1						
S3	−0.411**	0.533**	0.204**	0.166**	1					
S4	−0.287**	0.589**	0.122**	0.110**	0.100**	1				
SSRS	−0.348**	0.227**	0.182**	0.078*	0.126**	0.151**	1			
SS1	−0.411**	0.263**	0165**	0.154**	0.197**	0.135**	0.577**	1		
SS2	−0.160**	0.123**	0.105**	0.009	0.045	0.119**	0.729**	0.023	1	
SS3	−0.011	−0.021	0.035	−0.034	−0.04	−0.026	0.381**	0.016	−0.032	1

**Correlation is significant at the 0.01 level (2-tailed). *Correlation is significant at the 0.05 level (2-tailed).

MFROP-com, modified falls risk for older people in the community assessment; SAFE, self-awareness of falls in elderly scale; SSRS, the social support rating scale; S1, awareness of activity safety and environment; S2, awareness of physical functions; S3, awareness of medication; S4, awareness of cognitive behavior; SS1, subjective social support; SS2, objective social support; SS3, support utilization.

### 4.5 Factors associated with fall risk among community-dwelling older adults with functional limitations

As shown in Tables 4, 5, a multiple linear regression model was constructed to analyze the factors associated with MFROP - com scores among the study subjects (*n* = 825). The MFROP - com total score was used as the dependent variable, and demographic characteristics that were found to be significant in the univariate analysis were used as the independent variables. Disordered categorical variables, such as marital status and living arrangements, were incorporated into the model using dummy variable coding, with the first category serving as the reference group. The results indicated that the model was statistically significant (*F* = 74.145, *p* < 0.001) and accounted for approximately 53.6% of the variance in MFROP - com scores (adjusted *R*^2^ = 0.536, Table 5).

**Table 4:** Assignment of independent variables.

Variable	Assignment method
SAFE	Entered as continuous variable (original score)
Age	60 ~ 69 = 1; 70 ~ 79 = 2; ≥ 80 = 3
SSRS	Entered as continuous variable (original score)
Marital status	Married (0, 0, 0, 0); reference group; unmarried (0, 1, 0, 0); divorced (0, 0, 1, 0); widowed (0, 0, 0, 1)
Education	Primary school and below = 1; Secondary school = 2; High school = 3; College and above = 4
Living arrangement	Living with spouse (0, 0, 0, 0); reference group; living alone (0, 1, 0, 0); living with relatives (0, 0, 1, 0); living with care worker (0, 0, 0, 1)
Degrees of functional limitation	Slight functional limitation = 1; moderate functional limitation = 2; severe functional limitation = 3
Knowledge of fall prevention	Do not understand = 1; fair understanding = 2; understand = 3
Sleep quality	No sleep disorder = 1; sleep disorder = 2

SAFE, self-awareness of falls in elderly scale; SSRS, the social support rating scale.

**Table 5:** Multiple linear regression analysis for MFROP-com among participants (*n* = 825).

Variable	B	SE	*β*	*t*	*p*
SAFE	−0.463	0.021	−0.565	−21.803	<0.001**
SSRS	−0.422	0.054	−0.190	−7.757	<0.001**
Age	0.993	0.344	0.071	2.882	0.004
Degrees of functional limitation	−0.388	0.741	−0.014	−0.523	0.601
Knowledge of fall prevention	−0.556	0.347	−0.040	−1.602	0.110
Unmarried	2.630	0.804	0.088	3.270	0.001
Divorced	−0.432	0.756	−0.015	−0.571	0.568
Widowed	−0.427	0.723	−0.016	−0.590	0.555
Living alone	3.369	0.762	0.137	4.42	<0.001**
Living with relatives	2.687	0.755	0.110	3.559	<0.001**
living with care worker	2.781	0.821	0.099	3.387	0.001
Sleep quality	3.018	0.822	0.097	3.672	<0.001**

*R*^2^ = 0.543, Adjusted *R*^2^ = 0.536, *F* = 74.145.

**Correlation is significant at the 0.01 level (2-tailed); *Correlation is significant at the 0.05 level (2-tailed).

SAFE, self-awareness of falls in elderly scale; SSRS, the social support rating scale.

As shown in Table 5, multiple factors were identified as significant predictors of the MFROP-com score. Higher scores on the Self-awareness of Falls in Elderly (SAFE) scale (B = −0.463, *β* = −0.565, *p* < 0.001) and higher levels of social support, as measured by the Social Support Rating Scale (SSRS) (B = −0.422, *β* = −0.190, *p* < 0.001), were found to be associated with lower MFROP-com scores. Conversely, older age was a risk factor, correlating with higher MFROP-com scores (B = 0.993, *β* = 0.071, *p* = 0.004). Regarding living arrangements, all categories other than the reference group of “living with spouse” were significant predictors. Those living alone (B = 3.369, *β* = 0.137, *p* < 0.001), with relatives (B = 2.687, *β* = 0.110, *p* < 0.001), or with nursing workers (B = 2.781, *β* = 0.099, *p* = 0.001) had significantly higher MFROP-com scores. Other significant factors were being unmarried (B = 2.630, *β* = 0.088, *p* = 0.001) and having sleep disorders (B = 3.018, *β* = 0.097, *p* < 0.001); both were associated with higher MFROP-com scores. However, variables such as functional limitation level, fall safety knowledge, and educational attainment showed no statistically significant association with the outcome measure in this model (all *p* > 0.05).

## 5 Discussion

This cross-sectional study evaluated the fall risk among community-dwelling older adults with functional limitations in Western China. The mean total MFROP-com score was 26.51 ± 10.95, indicating a significant overall risk of falls (score > 12). This result falls within the range of risk levels reported in other high-risk older adults, such as those in long-term care facilities, community-dwelling older adults with diabetes, and hospitalized patients with chronic conditions (40–43). While direct comparisons are limited by differences in care settings, health status, and assessment methods, these references collectively underscore that elevated fall risk is a prevalent and serious issue among diverse groups of vulnerable older adults. Notably, the fall risk observed in this study was higher than that reported by Tang (44) involving older adults in first-tier cities. This discrepancy might be attributed to the targeted inclusion of functionally impaired older adults in the present study, who generally experience more severe frailty and functional decline, as well as regional disparities in community infrastructure, medical resources, and economic development between Western and more developed regions. Despite a high average risk, substantial individual variability (SD = 10.95) highlights the presence of a high-risk subgroup within this population that warrants prioritized intervention (45, 46). Evidence supports the effectiveness of functional exercises, such as Tai Chi and balance–resistance training, for fall prevention (47). For those with slight functional limitations, training focused on activities of daily living may be more appropriate. However, adherence is often compromised due to psychological resistance or reluctance to use assistive devices (12). Therefore, it is essential to leverage existing community resources—such as senior centers, neighborhood committees, and community health workers—to implement fall prevention education through both in-person and virtual formats (29). These efforts should aim to improve risk awareness, address cognitive biases, and promote the adoption of preventive behaviors.

Fall awareness is a significant independent predictor of fall risk among community-dwelling older adults with functional limitations, as confirmed by the present study. Multivariate linear regression analysis revealed a substantial negative correlation between SAFE scores and fall risk (B = −0.463, *β* = −0.565, *p* < 0.001), suggesting that enhancing cognitive vigilance for falls may be as effective as improving physical function in reducing the risk of falls (48, 49). Notably, the total SAFE scores in this study (57.89 ± 13.35) were higher than those reported for hospitalized older adults with stroke. This difference should be interpreted with caution, as it likely reflects a combination of factors. Distinct health statuses—such as post-acute stroke functional limitation in hospital settings versus community-dwelling functional limitation at home or in the community—may contribute to the observed SAFE score differences. Additionally, in hospitals, safety responsibilities are often delegated to staff, and the focus is on acute treatment, which can lower patients’ self-perceived risk, potentially affecting their SAFE scores (50). These comparisons highlight how both disease stage and living context distinctly influence fall awareness. Disease stage and living environment have a distinct influence on awareness levels. Further analysis of SAFE scores revealed a pattern of “highest awareness of medication and lowest awareness of physical functions,” consistent with findings from studies of rural older adults hypertensive patients (51). This highlights greater awareness of medication risks, frequently emphasized by healthcare providers, while gradual physical decline often goes underestimated due to habituation and cognitive normalization, creating potential blind spots in risk perception. These findings highlight the need for proactive initiatives that foster an objective self-awareness of physical capabilities in older adults, alongside continued medication safety education. At the practical level, this means guiding older adults to develop an objective understanding of their physical capabilities. This can be achieved through methods such as scenario-based workshops and videos of fall incidents, which aim to dispel the misconception that “I will not fall.” Furthermore, conducting home safety assessments can lead to targeted improvements, addressing environmental hazards like missing bathroom grab bars or inadequate nighttime lighting. Collectively, this approach shifts the focus from passive care to proactive risk prevention and control. This study identifies social support as an independent protective factor against fall risk in community-dwelling older adults with functional limitations (B = −0.422, *β* = −0.190, *p* < 0.001), a result consistent with Zhao who demonstrated that social support enhances psychological adaptability and behavioral regulation to reduce fall risk (51). Furthermore, correlation analysis revealed a significant negative association between total social support and fall risk (*r* = −0.348, *p* < 0.01), supporting Shear’s findings on the foundational role of social support in fall prevention among older adults (15). The protective mechanisms of social support operate through three distinct dimensions. First, subjective support (emotional care and respect) can alleviate anxiety and enhance confidence, thereby reducing fear-driven activity avoidance. Second, objective support (tangible assistance such as environmental modifications and help with daily activities) directly mitigates physical risks. Third, effective utilization of available support (e.g., accessing health education and skill training) improves adherence to preventive measures and enhances self-management efficacy (52, 53). Collectively, social support forms a multidimensional protective chain encompassing psychological, environmental, and behavioral pathways, underscoring the importance of systematically integrating family, community, and professional services to strengthen support networks and improve fall prevention in this vulnerable population.

This study indicates that age, sleep quality, living arrangement, and marital status are significant demographic factors influencing fall risk among community-dwelling older adults with functional limitations. Multiple linear regression indicated that older age was significantly associated with increased fall risk (B = 0.993, *β* = 0.071, *p* = 0.004). This association can be attributed to multisystem functional decline, which includes: (1) impaired balance control due to deficits in visual, vestibular, and proprioceptive systems; (2) gait abnormalities arising from sarcopenia; (3) elevated fracture risk from osteoporosis; and (4) diminished emergency response capacity due to neurocardiovascular degradation. These risks are particularly pronounced among adults over 80 years, whose markedly reduced physiological reserves heighten both care needs and fall susceptibility (10, 11, 54). Sleep disorders were also identified as an independent risk factor for falls (B = 3.018, *β* = 0.097, *p* < 0.001), since insufficient sleep compromises cognitive awareness, delays neuromuscular responses, and contributes to postural instability, thereby significantly increasing the likelihood of falls (55). Regarding living arrangements, older adults living alone (B = 3.369, *β* = 0.137, *p* < 0.001), living with relatives (B = 2.687, *β* = 0.110, *p* < 0.001), or living with care worker (B = 2.781, *β* = 0.099, *p* = 0.001) all demonstrated higher fall risk compared to those living with a spouse. The reasons for increased fall risk differ across living arrangement. Those living alone lack immediate support. Those living with relatives may face protection gaps due to caregivers’ limited skills or delayed responses. For residents in institutions, risks are heightened by unfamiliar environments—such as new room layouts, communal facilities, and structured routines—and by the functional challenge of navigating a non-personalized space with often-compromised physical abilities.53 Being unmarried was a significant risk factor (B = 2.630, *β* = 0.088, *p* = 0.001). This is primarily due to the absence of the marital “support reserve.” In practice, this means lacking the ongoing mutual support and monitoring that typically exists between spouses. Within a marriage, partners engage in shared risk management—for example, by reminding each other of hazards, assisting with challenging activities, and offering emotional reassurance. These interactions help regulate behavior, reduce anxiety, and address gaps in self-monitoring. Without this daily, interactive support system, fall risk may increase (56). To address these factors, we recommend implementing cognitive behavioral therapy combined with light therapy and physical exercise for older adults with sleep disorders to improve circadian rhythm; introducing smart monitoring technologies and strengthening community visit systems for those living alone; and prioritizing age-friendly environmental modifications and caregiver training in households with relatives to effectively reduce fall risk through multidimensional strategies.

## 6 Limitations

The findings of this study should be interpreted in the context of its limitations. Specifically, we employed a convenience sample of community-dwelling older adults with functional limitations from Western China, which may not be representative of older adults in other regions. Future research should aim to include older adults from various regions to gain a more comprehensive understanding of their fall risk and how to address these risks to reduce the likelihood of falls. Secondly, our primary measures (fall risk, fall awareness, social support) relied on self-reported questionnaires. While these are validated scales, responses may be subject to recall bias, social desirability bias, or individual differences in interpretation, which could introduce measurement error. Additionally, the cross-sectional nature of this research precluded causal inference between fall awareness, social support, and fall risk. Future studies would benefit from employing longitudinal designs and mixed-method approaches that incorporate more objective measures and qualitative insights to validate and deepen these findings.

## 7 Conclusion

This study surveyed 825 community-dwelling older adults with functional limitations and identified that the current fall risk was relatively high. Factors such as age, sleep quality, living arrangements, and marital status were found to be significant predictors of fall risk. Crucially, fall awareness and social support exert a significant influence on fall risk. These findings underscore the urgent need for multi-dimensional strategies to reduce fall risk and safeguard the well-being of this vulnerable population. We recommend developing integrated interventions among community-dwelling older adults with functional limitations that focus on the following: addressing misperceptions about fall risk through enhanced risk - perception education; cultivating robust support networks that involve families, communities, and health professionals; and implementing practical fall - prevention strategies such as caregiver training and age - friendly home modifications.

## Data Availability

The original contributions presented in the study are included in the article/supplementary material, further inquiries can be directed to the corresponding author.
